# *Streptomyces antimicrobicus* sp. nov., a novel clay soil-derived actinobacterium producing antimicrobials against drug-resistant bacteria

**DOI:** 10.1371/journal.pone.0286365

**Published:** 2023-05-31

**Authors:** Manee Chanama, Chanwit Suriyachadkun, Suchart Chanama

**Affiliations:** 1 Faculty of Public Health, Department of Microbiology, Mahidol University, Bangkok, Thailand; 2 Thailand Bioresource Research Center (TBRC), National Center for Genetic Engineering and Biotechnology (BIOTEC), National Science and Technology Development Agency (NSTDA), Pathum Thani, Thailand; 3 Faculty of Science, Department of Biochemistry, Chulalongkorn University, Bangkok, Thailand; Universidade de Coimbra, PORTUGAL

## Abstract

A novel actinobacterium, designated strain SMC 277^T^, was isolated from the clay soil in paddy field of Chonburi Province, Thailand, and characterized using polyphasic taxonomy. Strain SMC 277^T^ formed straight chains of nonmotile cylindrical spores with smooth surface developed on aerial mycelia. The typical chemotaxonomic properties of members of the genus *Streptomyces* were observed in strain SMC 277^T^, e.g., cell wall peptidoglycan, whole cell sugars, major menaquinones, cellular fatty acids, and polar lipids. Chemotaxonomic data combined with mycelium and spore morphologies supported the assignment of strain SMC 277^T^ to the genus *Streptomyces*. The results of comparative analysis of the 16S rRNA gene sequences confirmed that strain SMC 277^T^ represented a member of the genus *Streptomyces*. Phylogenetic analysis based on 16S rRNA gene sequences indicated that strain SMC 277^T^ shared the highest sequence similarity with *Streptomyces bambusae* NBRC 110903^T^ (98.8%). Genome sequencing revealed a genome size of 6.55 Mbp and a digital G+C content of 73.4 mol%. In addition to the differences in phenotypic characteristics (morphology and physiology), values of ANI (ANIb and ANIm), AAI and dDDH between strain SMC 277^T^ and its closest relative *S*. *bambusae* NBRC 110903^T^ were 81.84, 86.77, 76.91 and 26.1%, respectively. Genome annotation and secondary metabolite gene cluster analysis predicted that SMC 277^T^ contained 35 biosynthetic gene clusters encoding diverse bioactive secondary metabolites. It is in agreement with observed antimicrobial activity against drug-resistant bacteria associated with nosocomial infections (methicillin-resistant *Staphylococcus aureus*, extended-spectrum β-lactamase producing *Klebsiella pneumoniae*, and multidrug-resistant *Acinetobacter baumannii*). On the basis of these genotypic and phenotypic characteristics, strain SMC 277^T^ can be characterized to represent a novel species of the genus *Streptomyces*, for which the name *Streptomyces antimicrobicus* is proposed. The type strain is SMC 277^T^ (= TBRC 15568^T^ = NBRC 115422^T^).

## Introduction

The genus *Streptomyces* classified in the family *Streptomycetaceae* of the suborder *Streptomycineae* [1,2] was proposed by Waksman and Henrici [3], and emended subsequently by Rainey et al. [4] and Kim et al. [5]. It is the largest genus of the *Actinobacteria* with 699 validly published and correct species, based on the LPSN (List of Prokaryotic names with Standing in Nomenclature, https://lpsn.dsmz.de/genus/streptomyces) website at the time of writing. Members of the genus *Streptomyces* are identified as aerobic, gram-stain-positive, non-acid-fast bacteria that form extensively branched substrate and aerial mycelia. They have long chains of spores, contain LL-diaminopimelic acid in their cell walls, major amounts of saturated, iso-and anteiso-fatty acids, and typically possess either MK-9(H_6_) or MK-9(H_8_) menaquinones. The predominant polar lipids contain diphosphatidylglycerol, phosphatidylethanolamine, phosphatidylinositol and phosphatidylinositol mannoside. The high genomic DNA G+C contents (66–78 mol%) are observed [6].

Obviously, *Streptomyces* strains are promising resources to produce bioactive compounds as antibiotics [7–10]. Several novel bioactive compounds with diverse antimicrobial activities have been reported from various species of the genus *Streptomyces* isolated from soils in different areas, for example, albocycline-type macrolides [11], benditerpenoic acid [12], and pyrimidomycin [13]. Until now, bacteria causing nosocomial infections still remain public health crisis and health security threats due to increasing antibiotic resistance [14]. Therefore, discovering novel *Streptomyces* species with potential bioactivities is crucial.

In this study, a polyphasic taxonomy, including morphological, physiological, chemotaxonomic and genotypic characterization was conducted to classify a novel actinomycete strain, designated SMC 277^T^, isolated from the clay soil in paddy field of Chonburi Province, Thailand. The inhibitory activities against drug-resistant bacteria of nosocomial infections, and biosynthetic potential of the strain were also investigated. Strain SMC 277^T^ represents a novel species of the genus *Streptomyces*, for which the name *Streptomyces antimicrobicus* sp. nov. is proposed.

## Materials and methods

### Isolation and culture conditions

During an investigation of actinomycete diversity from soils in Thailand, strain SMC 277^T^ was isolated from a sample of clay soil collected from a paddy field of Chonburi Province. The sample was kept at -20°C before being air-dried at 37°C for 7 days. The strain was isolated using the standard dilution plate method and grown on humic acid-salts vitamin agar [15] supplemented with cycloheximide (50 mg/L) and nystatin (50 mg/L). After incubating at 28°C for 7 days, the colony of strain SMC 277^T^ was selected and then subcultivated on International *Streptomyces* Project (ISP) 3 medium (oatmeal agar) [16]. A pure culture was maintained in glycerol (20%, v/v) at -80°C. The type strain of the closest species, *Streptomyces bambusae* NBRC 110903^T^ was obtained from Thailand Bioresource Research Center (TBRC). The strain was cultured under the same conditions, and used to compare polyphasic characteristics.

### Morphology

Morphological characterization of strain SMC 277^T^ and the type strain, *Streptomyces bambusae* NBRC 110903^T^, was performed after growth on various International *Streptomyces* Project (ISP) media (ISP 2–7), as described by Shirling and Gottlieb [17], after incubating at 28°C for 14 days. The Inter-Society Color Council National Bureau of Standards (ISCC-NBS) color charts [18] were used to determine the color of aerial and substrate mycelium, and soluble pigments. Morphology of mycelia and spores was observed after cultivating on modified soil extract agar [19] at 28°C for 10 days under both a light microscope (model CX31, Olympus, Japan) with a x50 working distance objective lens (model SLMPLN50X, Olympus, Japan) and a scanning electron microscope (SEM) (model JSM-IT500HR, JEOL, Japan). The sample for SEM was prepared by fixing an agar block on which the culture grew with 2% osmium tetroxide vapor, followed by dehydration through a graded ethanol series, and finally dried using a critical point dryer (Leica model EM CPD300, Austria). The dried sample was placed on the stub and coated with gold using sputter coater (Balzers model SCD040, Germany) for visualization under SEM.

### Physiology and biochemical properties

Growth at different temperatures (20, 25, 30, 37, 40 and 45°C) was assessed on ISP 2 medium after incubating at 28°C for 14 days. The pH range at pH 4.0 to 11.0 (intervals of 1.0 pH unit) and NaCl tolerance with 0.5, 1, 2, 3, 4 and 5% (w/v) for growth were determined using ISP 2 medium as a basal medium after 14 days of incubating at 28°C. Using carbohydrates as a sole carbon source was examined on ISP 9 medium as a basal medium supplemented with a final concentration of 1% (w/v) of the carbon sources [20]. The hydrolysis of various substrates was evaluated using a basal medium and method recommended by Gordon *et al*. [21]. Gelatin liquefaction, milk peptonization and coagulation, nitrate reduction and hydrolysis of starch, xanthine and hypoxanthine were determined by cultivating on various media as described by Arai [22], and Williams and Cross [23]. Enzyme activities were examined using the API-ZYM system (bioMérieux) according to the manufacturer instructions.

### Chemotaxonomic analysis

Biomass of strain SMC 277^T^ and its closest relative, *S*. *bambusae* NBRC 110903^T^ used for chemotaxonomic analysis was obtained from culture grown in glucose-yeast extract broth [24] on a rotary shaker at 28°C, 250 rpm for 7 days. Cells were harvested using centrifugation, washed three times with sterile distilled water before freeze drying. The isomer of diaminopimelic acid in the cell wall was determined using the method of Staneck and Roberts [25]. The compositions of reducing sugar in whole-cell hydrolysates were analyzed using cellulose TLC as described by Komagata and Suzuki [26]. Total polar lipids in whole cells were extracted and analyzed according to the method of Minnikin *et al*. [27]. Cellular fatty acids were prepared and analyzed following the instructions of the RTSBA6 method of the Microbial Identification System (MIDI; Sherlock, Version 6.4) [28]. Menaquinones were extracted and purified using the method of Collins et al. [29], and analyzed using reverse-phase HPLC [Cosmosil 5C_18_ column (4.6x150 mm); Nacalai Tesque] with a mixture of methanol and 2-propanol (2:1, v/v) as elution solvent [30].

### Genomic and phylogenetic characterization

Genomic DNAs of strain SMC 277^T^ and *Streptomyces bambusae* NBRC 110903^T^ were extracted according to a modified method of Saito and Miura [31] from cells grown in glucose-yeast extract broth at 28°C, 250 rpm for 5 days. Freeze-dried cells were lysed by grinding with mortar and pestle, instead of lysozyme. The 16S rRNA gene of strain SMC 277^T^ was amplified using PCR with 27F and 1492R primers as described by Monciardini *et al*. [32]. The amplified 16S rRNA gene was purified and directly sequenced by SolGent (Seoul, South Korea) using the ABI373OXL platform and 4 sequencing primers (2F: 5′ ACGGGAGGCAGCAGTG 3′, 3F: 5′ AACACCGGTGGCGAAG 3′, 4F: 5′ CGTCAAGTCATCATGCCC 3′, 4R: 5′ CCTACGWGCYCTTTACGCC 3′). The nucleotide sequences obtained from all primers were assembled using the Cap contig assembly program, an accessory application in the Bioedit Sequence Alignment Editor Software (Version 7.2.5) [33]. The Basic Local Alignment Search Tool (BLAST) analysis retrieved from the nucleotide databases of NCBI (https://blast.ncbi.nlm.nih.gov/Blast.cgi; [34] was used to compare the almost-complete 16S rRNA gene sequence of strain SMC 277^T^ with sequences of all validly published species of the genus *Streptomyces*. Based on pairwise alignment using the NCBI BLAST database [34], the 16S rRNA gene sequence similarities between species were calculated. Multiple alignments were carried out using the CLUSTAL W Program [35] in Bioedit Sequence Alignment Editor Software (Version 7.2.5) [33]. *Nocardioides albus* KCTC 9186^T^ was taken as an outgroup. The MEGA Software Package, Version 10.1.7 [36] was used to construct a phylogenetic tree using neighbor-joining [37], maximum-parsimony [38], and maximum-likelihood [39] methods. The neighbor-joining result was calculated according to Kimura’s two-parameter model with complete deletion. The search method for maximum-parsimony was subtree-pruning-regrafting. The Tamura 3-parameter plus gamma distributed with invariant sites was used for maximum-likelihood. The statistical reliability of the tree topology was evaluated using bootstrap analysis with 1000 replications [40].

Whole genomes of strain SMC 277^T^ and *S*. *bambusae* NBRC 110903^T^ were sequenced using Illumina NovaSeq platform at Novogene (Beijing, China). Libraries of genomic DNA were prepared using a TruSeq Nano DNA kit (Illumina) and pooled libraries were subjected to multiplexed paired-end sequencing. Raw paired-end sequences were used for quality control, and their adapter and primer sequences were trimmed using the FASTP Program [41]. The cleaned sequences were then used to assemble the genome with SPAdes (Version 3.10.1) [42]. The assembled genome was annotated using the NCBI Prokaryotic Genome Annotation Pipeline [43–45]. The average nucleotide identity (ANI) values, i.e. ANI-BLAST (ANIb) and ANI-MUMmer (ANIm) of the whole genome of strain SMC 277^T^ with closely related type strains were calculated using the JSpeciesWS web server [46]. The average AAI of strain SMC 277^T^ compared with closely related type strains was calculated using the online server AAI calculator (http://enve-omics.ce.gatech.edu/aai/) [47]. The Type (Strain) Genome Server (TYGS), a free bioinformatics platform available at https://tygs.dsmz.de, was used to analyze the whole genome-based taxonomic [48]. The tree was inferred using FastME 2.1.6.1 [49] from the Genome BLAST Distance Phylogeny Approach (GBDP) distances calculated from genome sequences, and branch support was inferred from 100 pseudo-bootstrap replicates. Digital DNA-DNA hybridization (dDDH) values between strain SMC 277^T^ and each of the closely related type strains was calculated using the recommended settings of the Genome-to-Genome Distance Calculator (GGDC), Version 2.1 of TYGS [48,50]. The genomic DNA G+C content (mol%) was determined from the whole genome data sequencing.

### Genome annotation and secondary metabolite gene cluster analysis

Genome assemblies of strain SMC 277^T^, and closely related type strains, including *S*. *bambusae* NBRC 110903^T^, *S*. *toxytricini* NBRC 12823^T^, *S*. *cirratus* NBRC 13398^T^, *S*. *vinaceus* ATCC 27476^T^, *S*. *nojiriensis* JCM 3382^T^, *S*. *yangpuensis* DSM 100336^T^, *S*. *virginiae* NBRC 12827^T^, and *S*. *amritsarensis* MTCC 11845^T^ were downloaded from NCBI. Protein-coding sequences (CDS) were predicted using Prodigal 2.6.3 [51]. Subsequently, all the annotated protein sequences were grouped to clusters of orthologous groups (COGs) using WebMGA [52]. The predicted secondary metabolite biosynthesis gene clusters were obtained using antiSMASH [53], and BLAST known cluster function was enabled to find known substances matched from repository of known biosynthetic gene clusters. ClustVis [54], a tool for hierarchical clustering, was used to create heatmaps and visualize differences of the COGs and biosynthesis gene clusters among *Streptomyces* species.

### Antimicrobial bioassays

Target microorganisms for antimicrobial bioassays were bacteria associated with nosocomial infections, called ESKAPE pathogens (*Enterococcus faecalis*, *Staphylococcus aureus*, *Klebsiella pneumoniae*, *Acinetobacter baumannii*, *Pseudomonas aeruginosa*, and *Enterobacter aerogenes*). Drug-sensitive bacteria, i.e., *E*. *faecalis* DMST 2860, *S*. *aureus* DMST 8840, *K*. *pneumoniae* DMST 7592, *A*. *baumannii* DMST 10437, *P*. *aeruginosa* DMST 4739 and *E*. *aerogenes* DMST 8841, were purchased from the National Institute of Health of Thailand, whereas drug-resistant bacteria, i.e., methicillin-resistant *Staphylococcus aureus* (MRSA) AMH 10, extended-spectrum β-lactamase (ESBL) producing *Klebsiella pneumoniae* AMH 20 and multidrug-resistant (MDR) *Acinetobacter baumannii* AMH 30, were kindly provided by Ananda Mahidol Hospital (AMH), Lopburi Province, Thailand.

Strain SMC 277^T^ was precultured in a 125 ml flask containing 10 ml of SCM medium [55] for 3 days at 28°C with 250 rpm shaking. Each 1% (v/v) of the seed culture was then transferred to each 250 ml flask containing 50 ml of SCM medium, and incubated at 28°C, 250 rpm for 7, 14 and 21 days separately. Each supernatant was added with 5 ml of Diaion^®^ HP-20 resin (Sigma). After leaving the suspension for 2 h under shaking, the resin was washed with 25 ml of water, filtered, and eluted with 25 ml of 80% methanol. The methanolic extract was evaporated to dryness, and dissolved with 100 μl of 67% methanol. Each 5 μl of the extract was transferred onto a paper disk (diameter of 6 mm, Whatman) to examine bioactivity. For bacterial targets, single colonies grown on tryptic soy agar (Becton Dickinson) at 37°C overnight were picked, adjusted to 10^8^ CFU/ml with tryptic soy broth (Becton Dickinson) and swabbed on the entire surface of Muller Hinton agar (MHA) (Becton Dickinson). The paper disk with extract was placed on the surface of MHA plates containing the target microorganisms. A disk with 5 μl of 67% methanol was used as a negative control. The plate was incubated at 37°C for 24 h and then measured for the diameter of zone of inhibition. Each test was performed in triplicate.

## Results and discussion

### Phylogenetic analysis based on 16S rRNA genes

Strain SMC 277^T^ isolated from the clay soil in paddy field collected from Chonburi Province, Thailand was used to characterize the taxonomic status using polyphasic approaches. The 16S rRNA gene is generally considered to be highly conserved, and can be used to identify prokaryotes at the genus and species level which are evaluated at 97.0 and 98.7%, respectively [56]. Firstly, the almost-complete 16S rRNA gene sequence of strain SMC 277^T^ (1476 bp, Genbank accession number OK380049) was obtained from the amplification of its gene using PCR, and analyzed to compare nucleotide sequence similarity with sequences of currently and validly published type strains using NCBI BLAST. The pairwise alignments of strain SMC 277^T^ showed the sequence similarity to those of members of genus *Streptomyces* with the top 41 *Streptomyces* spp. ranking from 98.8–97.6%, e.g., *Streptomyces bambusae* NBRC 110903^T^ (98.8%), *S*. *griseocarneus* DSM 40004^T^ (98.4%), *S*. *coerulescens* NBRC 12758^T^ (98.3%), *S*. *abikoensis* NBRC 13860^T^ (98.2%), *S*. *yangpuensis* DSM 100336^T^ (98.2%). The sequence similarity of the strain SMC 277^T^ was the most matched to that of *Streptomyces bambusae* NBRC 110903^T^ (98.8%), which this value was slightly higher than defined value of species delineation (98.7%) **for determining the bacterial strains to the same genomic species [57]. Hence, this result** provides us a clue to predict that SMC 277^T^ has genetic differences from any other type strains of species of the genus *Streptomyces*, and may represent a novel species of the genus.

**Fig 1:**
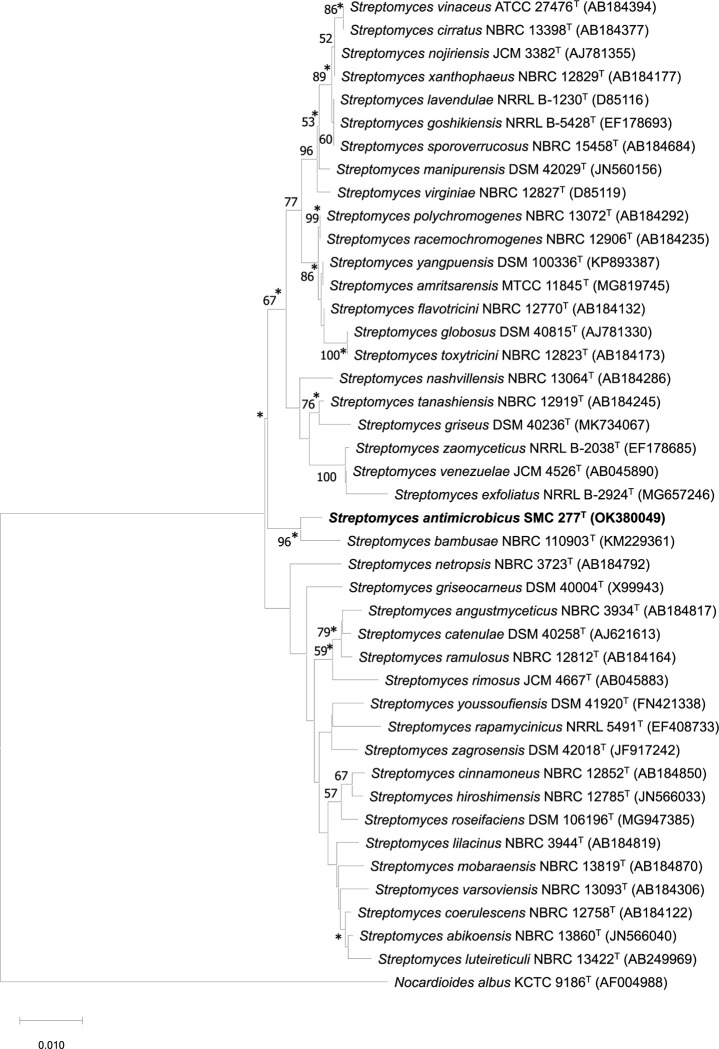
Phylogenetic relationships based on neighbor-joining analysis of 16S rRNA gene sequence (1476 nt) of strain SMC 277^T^ and the top 41 closely related members of the genus *Streptomyces* obtained from the NCBI BLAST database. *Nocardioides albus* KCTC 9186^T^ was used as an outgroup. Asterisks (*) indicate the branches of the tree that were found using maximum-parsimony and maximum-likelihood methods. The numbers on the branches indicate the percentage bootstrap values of 1,000 replicates, and only values >50% are shown. Bar 0.010 substitutions per nucleotide position.

Moreover, the phylogenetic trees reconstructed by all algorithms of neighbor-joining (Fig 1), maximum-parsimony (S1A Fig) and maximum-likelihood (S1B Fig) yielded similar topology indicating that SMC 277^T^ formed a monophyletic clade with only *S*. *bambusae* NBRC 110903^T^, with high bootstrap support at 96, 92 and 97%, respectively. For this reason, *S*. *bambusae* NBRC 110903^T^ was selected as the closest phylogenetic relative for comparative analysis. Even though the results of phylogenetic relationship exhibited that strain SMC 277^T^ belonged to the genus *Streptomyces* with *S*. *bambusae* NBRC 110903^T^ as the closest relative, overall genomic relatedness indices (ORGIs) should be further calculated between genome sequences of strain SMC 277^T^ and closely related type strains in order to delineate SMC 277^T^ as a new species.

### Genome features and genotypic analysis

It has been proven that the information of genome sequences is objective and reliable for the prokaryotic taxonomy [57]. The overall genomic relatedness indices (OGRIs) including average nucleotide identity (ANI), average amino acid identity (AAI) and digital DNA-DNA hybridization (dDDH) can be used to examine if a strain belongs to a known species. Therefore, OGRI values should be combined with 16S rRNA gene sequence similarity for a systematic process to identify and recognize a new species [57].

**Fig 2:**
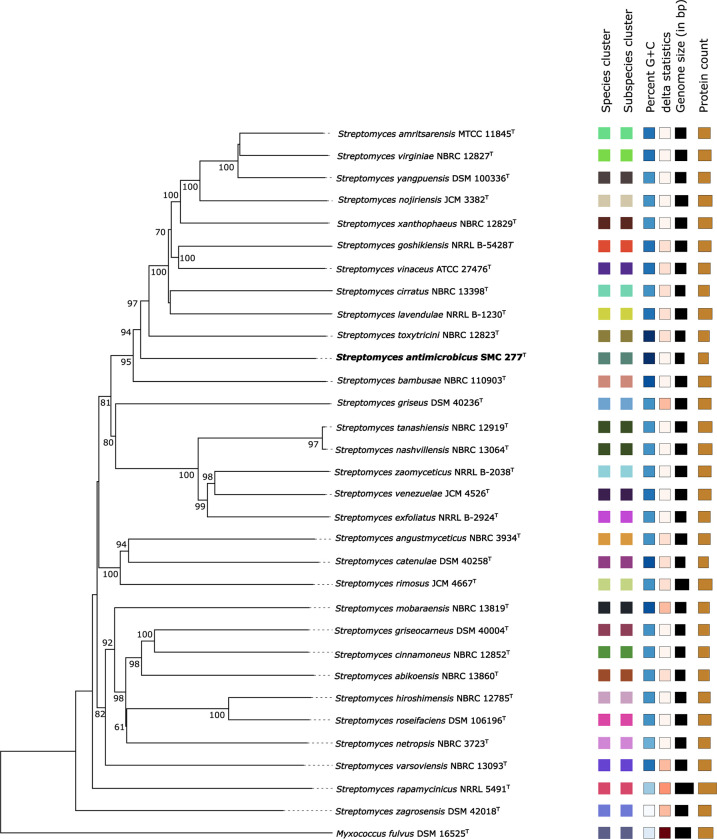
Phylogenomic tree based on genome sequences of strain SMC 277^T^ in the TYGS server. *Myxococcus fulvus* DSM 16525^T^ was used as an outgroup. Tree inferred with FastME 2.1.6.1 [49] from GBDP distances calculated from genome sequences. The branch lengths are scaled in terms of GBDP distance formula *d*_*5*_. The numbers above branches are GBDP pseudo-bootstrap support values >60% from 100 replications, with an average branch support of 85.3%. The tree was rooted at the midpoint [59].

On the basis of genome sequencing, the draft genomes of strain SMC 277^T^ (284 contigs), and *S*. *bambusae* NBRC 110903^T^ (169 contigs) have been submitted to GenBank with accession numbers of JAJAUY000000000 and JAJAUZ000000000, respectively, and are publicly available. The draft genome sequence of strain SMC 277^T^ yielded the size of 6.55 Mbp with an average in silico DNA G+C content of 73.4 mol%, and total of 5,809 protein-CDSs and 78 RNAs, whereas the genome size of *S*. *bambusae* NBRC 110903^T^ was 8.31 Mbp with DNA G+C content of 73.0 mol%, and total of 7,269 CDSs and 75 RNAs. To further clarify the relationship between strain SMC 277^T^ and closely related type strains, we obtained genomic data for 30 of the 41 strains with the highest 16S rRNA gene sequence similarity to strain SMC 277^T^ (S1 Table). The whole genome phylogeny of strain SMC 277^T^ indicated that it constituted a member of the genus *Streptomyces*, and was clearly separated from *S*. *bambusae* NBRC 110903^T^ (Fig 2). Both average nucleotide identity (ANI) values, namely, ANIb and ANIm, of strain SMC 277^T^ and *S*. *bambusae* NBRC 110903^T^ were 81.84% and 86.77%, respectively. The average amino acid identity (AAI) value of strain SMC 277^T^ with *S*. *bambusae* NBRC 110903^T^ was 76.91%. The digital DNA-DNA hybridization (dDDH) value between the genomes of strain SMC 277^T^ and *S*. *bambusae* NBRC 110903^T^ was 26.1% (C.I. model 23.7 to 28.6%). The ANI (ANIb and ANIm), AAI and dDDH values of strain SMC 277^T^ and other closely related species exhibited in the phylogenomic tree were shown in S1 Table. Clearly, these ANI, AAI and dDDH values were below the thresholds of 95, 95 and 70%, respectively, for prokaryotic species delineation [57,58]. The results of OGRIs (ANI, AAI and dDDH), and phylogenomic relationship were sufficient to categorize strain SMC 277^T^ as representing a distinct species from previously described *Streptomyces* species.

### Morphological and physiological characteristics

**Fig 3:**
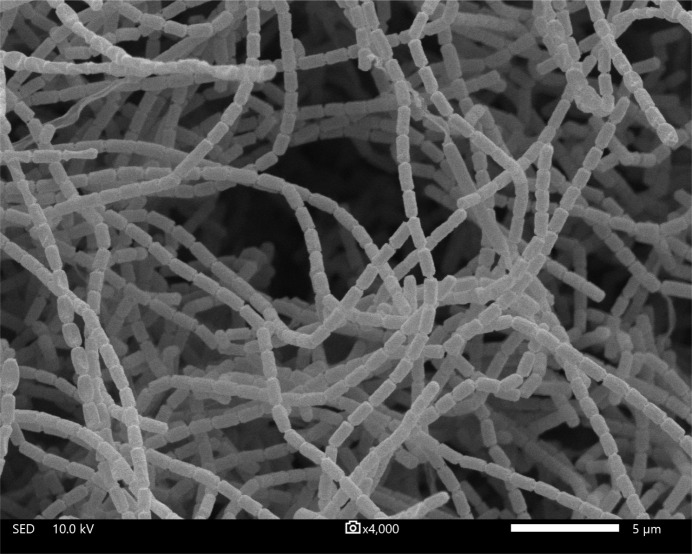
Scanning electron micrograph of strain SMC 277^T^. The strain exhibited straight spore chains with smooth spore surfaces on its aerial mycelia. It was grown on modified soil extract agar at 28°C for 10 days. Bar, 5 μm.

To support the novelty at the species level of SMC 277^T^, phenotypic characteristics including morphology and physiology were performed. It developed straight spore chains on only aerial mycelium. Each spore was smooth-surfaced, and measured 0.5 to 0.7 by 1.1 to 1.3 μm in size (Fig 3). The mycelium and spore morphologies of SMC 277^T^ were consistent with members of the genus *Streptomyces*. Strain SMC 277^T^ exhibited good growth on ISP 2 and ISP 3 media, moderate growth on ISP 4 media, and poor growth on ISP 5, ISP 6 and ISP 7 media. The color of the substrate mycelium on these media was pale yellow to grayish greenish yellow. No aerial mycelium was produced on ISP 6, while the aerial mycelium with yellowish white to pinkish gray was produced on other growth media. The diffusible pigment was not observed on all tested media (S2 Table). The phenotypic comparison between SMC 277^T^ and the closest relative, *S*. *bambusae* NBRC 110903^T^ revealed differential characteristics that enabled SMC 277^T^ to be readily distinguished from the closest relative (Table 1). Unlike *S*. *bambusae* NBRC 110903^T^, strain SMC 277^T^ was able to grow at the maximum temperature of 40°C. Even though SMC 277^T^ and *S*. *bambusae* NBRC 110903^T^ were capable of growing at the maximum tolerance to NaCl of 4% (w/v), the growth capability of SMC 277^T^ was higher than that of *S*. *bambusae* NBRC 110903^T^ at 0.5, 1 and 2%. Moreover, hydrolysis of xanthine, gelatin liquefaction, nitrate reduction, milk coagulation and peptonization, the use of D-arabinose, cellobiose, D-galactose, D-mannitol, D-trehalose and D-xylose as sole carbon sources, and the capabilities to produce various enzymes, such as alkaline phosphatase, cystine arylamidase, α-glucosidase and α-mannosidase were the apparent characteristics for separating between SMC 277^T^ and *S*. *bambusae* NBRC 110903^T^. Additionally, morphological and physiological characteristics of strain SMC 277^T^ and other closely related *Streptomyces* species showing 16S rRNA similarity values below cut-off point of 98.7%, including *S*. *griseocarneus* DSM 40004^T^, *S*. *coerulescens* NBRC 12758^T^, *S*. *abikoensis* NBRC 13860^T^, *S*. *yangpuensis* DSM 100336^T^, *S*. *virginiae* NBRC 12827^T^, *S*. *amritsarensis* MTCC 11845^T^, *S*. *toxytricini* NBRC 12823^T^ and *S*. *cirratus* NBRC 13398^T^ were also presented in S2 Table. These informative data have been supporting evidence for phenotypic differences between SMC 277^T^ and each of these closely related type strains. Therefore, strain SMC 277^T^ could be distinguished from other *Streptomyces* taxa, and represented as a novel species.

**Table 1:** Phenotypic characteristics that distinguish *Streptomyces* sp. SMC 277^T^ from the most closely related type strain, *Streptomyces bambusae* NBRC 110903^T^.

Characteristics	1	2
Color of substrate mycelium on		
ISP 2	Pale Yellow	Grayish Yellow
ISP 3	Pale Yellow	Grayish Yellow
ISP 4	Pale Orange Yellow	Grayish Yellow
ISP 5	Yellowish White	Grayish Yellow
ISP 6	Grayish Greenish Yellow	Dark Greenish Gray
ISP 7	Yellowish White	Light Olive Brown
Growth temperature at (°C)	20–40	20–30
Growth with NaCl (%w/v)		
0.5%	+	w
1%	+	w
2%	+	w
Hydrolysis of xanthine	+	-
Gelatin liquefaction	-	+
Milk coagulation	+	-
Milk peptonization	-	+
Nitrate reduction	-	+
Use of:		
D-Arabinose	+	w
Cellobiose	-	+
D-Galactose	-	w
D_Mannitol	-	w
D-Trehalose	w	-
D-Xylose	-	+
Enzyme activity:		
Alkaline phosphatase	+	w
Cystine arylamidase	w	-
α-Glucosidase	+	-
α-Mannosidase	+	-

Strains: 1, SMC 277^T^; 2, *S*. *bambusae* NBRC 110903^T^. All data were obtained in this study. +, positive; -, negative; w, weakly positive. All strains were positive for D-glucose, D-maltose, D-mannose, glycerol and sucrose, negative for arabitol, D-fructose, D-lactose, D-melibiose, D-raffinose, D-ribose, L-rhamnose, L-sorbose, inositol, sorbitol and xylitol, and weakly positive for salicin.

### Chemotaxonomic characteristics

Chemotaxonomy is also one of key information for describing a new taxon. Hence, these characteristics were investigated to confirm the taxonomic affiliation of strain SMC 277^T^ at the genus level. Strain SMC 277^T^ exhibited chemotaxonomic characteristics that were typical profiles of the genus *Streptomyces*. Strain SMC 277^T^ contained LL-diaminopimelic acid in the cell wall peptidoglycan (S2 Fig), which was a common feature of members of the genus *Streptomyces* [6]. Whole cell sugars consisted of glucose, galactose, mannose and ribose (S3 Fig). The major menaquinones were detected as MK-9(H_8_) (79.5%) and MK-9(H_6_) (20.5%). The major menaquinones of SMC 277^T^ were similar to those found in the closest relative, *S*. *bambusae* NBRC 110903^T^, with different proportions [60]. Polar lipids consisting of phosphatidylglycerol, diphosphatidylglycerol, phosphatidylethanolamine, phosphatidylinositol, phosphatidylinositol mannoside and phospholipids were detected in the cells (S4 Fig). The predominant cellular fatty acids (>10%) were iso-C_16:0_ (26.4%), anteiso-C_15:0_ (20.9%) and anteiso-C_17:0_ (10.8%) (Table 2). The pattern of major fatty acids in the cells of SMC 277^T^ was similar to those predominant fatty acids in *S*. *bambusae* NBRC 110903^T^, the closest relative with different proportions (Table 2). Therefore, the chemotaxonomic analysis confirmed that strain SMC 277^T^ represented a member of the genus *Streptomyces*.

**Table 2:** Cellular fatty acid profiles of *Streptomyces* sp. SMC 277^T^ and the closest relative, *Streptomyces bambusae* NBRC 110903^T^.

Fatty acids	1	2
**Saturated fatty acids**		
C_16:0_	5.9	8.8
C_17:0_ cyclo	3.0	2.7
C_18:0_ 3-OH	-	1.6
**Branched fatty acids**		
Iso-C_14:0_	6.3	5.0
Iso- C_15:0_	6.4	6.6
Anteiso-C_15:0_	**20.9**	**24.6**
Iso-C_16:0_	**26.4**	**20.1**
Iso-C_17:0_	3.9	4.1
Anteiso-C_17:0_	**10.8**	**9.7**
Anteiso-C_17:1_ ω9c	3.27	2.43
Iso-C_18:0_	1.1	<1
**Unsaturated branched fatty acids**		
Iso-C_16:1_ H	3.8	1.5
Iso-C_18:1_ H	1.0	<1
Summed feature 3^a^	2.3	5.4
Summed feature 9^b^	1.1	1.7

Strain: 1, SMC 277^T^; 2, *S*. *bambusae* NBRC 110903^T^. All data were from this study. Values are expressed as percentages of total fatty acids. Fatty acids of all of four strains are undetected or yield lower than 1% are omitted. ^_^, undetected.

^a^Summed feature 3 comprised C_16:1_ ω7c and/or C_16:1_ ω6c.

^b^Summed feature 9 comprised C_16:0_ 10-methyl and/or iso-C_17:1_ ω9c.

### Genome annotation and secondary metabolite gene cluster analysis

**Fig 4:**
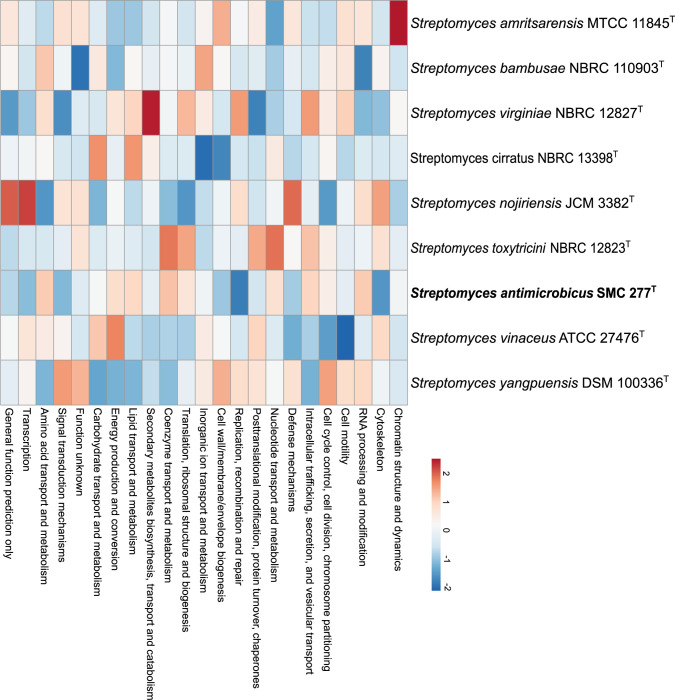
Hierarchical clustering of heatmap showing differences between functional classification of clusters of orthologous groups present in the genomes of strain SMC 277^T^ and closely related type strains. Heatmap shows a data matrix where coloring gives an overview of the numeric differences from a reference.

A total of 23 functional categories of COG proteins were observed in genomes of strain SMC 277^T^, the closest relative, *S*. *bambusae* NBRC 110903^T^, and other closely related type strains in the same phylogenomic clade as SMC 277^T^, including, *S*. *toxytricini* NBRC 12823^T^, *S*. *cirratus* NBRC 13398^T^, *S*. *vinaceus* ATCC 27476^T^, *S*. *nojiriensis* JCM 3382^T^, *S*. *yangpuensis* DSM 100336^T^, *S*. *virginiae* NBRC 12827^T^ and *S*. *amritsarensis* MTCC 11845^T^. The three most abundant proteins were associated with general cellular function, transcription and amino acid transport and metabolism, respectively (S3 Table). In addition, hierarchical clustering of heatmap (Fig 4) showed different COG proteins clustered in different strains. In fact, strain SMC 277^T^, *S*. *bambusae* NBRC 110903^T^, *S*. *vinaceus* ATCC 27476^T^, *S*. *cirratus* NBRC 13398^T^ and *S*. *toxytricini* NBRC 12823^T^ were clustered in a group having a widespread abundance of COG proteins, whereas *S*. *virginiae* NBRC 12827^T^, showed enrichment in inorganic ion transport and metabolism; coenzyme transport and metabolism; translation, ribosomal structure and biogenesis; intracellular trafficking, secretion and vesicular transport; energy production and conversion; amino acid transport and metabolism; carbohydrate transport and metabolism; lipid transport and metabolism; and secondary metabolite biosynthesis and transport and catabolism. On the other hand, *S*. *yangpuensis* DSM 100336^T^, *S*. *amritsarensis* MTCC 11845^T^ and *S*. *nojiriensis* JCM 3382^T^ showed enrichment in general function prediction; transcription; defense mechanisms; cell cycle control, cell division, chromosome partitioning; signal transduction mechanisms; posttranslational modification, protein turnover, chaperones; RNA processing and modification; and unknown function.

**Fig 5:**
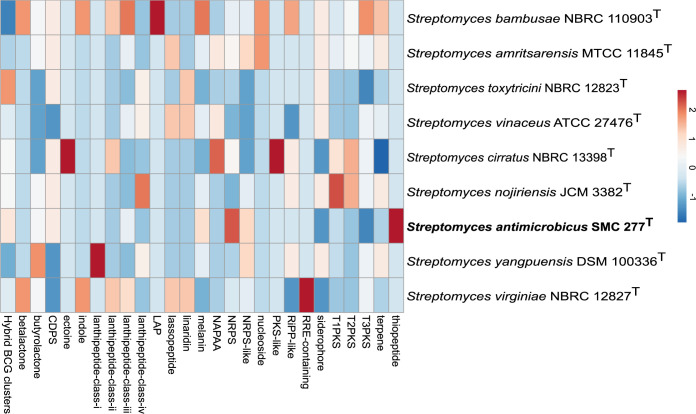
Hierarchical clustering of heatmap showing differences between type of biosynthetic gene clusters predicted by antiSMASH in the genomes of strain SMC 277^T^ and closely related type strains. Heatmap shows a data matrix where coloring gives an overview of the numeric differences from a reference.

Genomes of strain SMC 277^T^ and closely related type strains mentioned as above were also scanned with antiSMASH to explore the known and putative secondary metabolite biosynthetic potential in their genome sequences. Altogether, 27 types of secondary metabolites and a total of 285 secondary metabolite biosynthesis gene clusters were identified (Fig 5 and S4 Table). The average number of BGCs of all *Streptomyces* strains was 32. The most abundant BGCs were hybrid clusters followed by terpene. However, *S*. *antimicrobicus* SMC 277^T^ exhibited 35 gene clusters with NRPSs as the dominant classes of predicted BGCs. Interestingly, by comparing secondary metabolite clusters and their products from the antiSMASH database among all *Streptomyces* strains, *S*. *antimicrobicus* SMC 277^T^ revealed 7 BCGs showing less than 50% similarity to known BCGs, including guadinomine, totopotensamide, SCO-2138, JBIR-78, griseoviridin, salinamide A and indigoidine. These BCGs were not present in any of the other analyzed strains. This suggested that *S*. *antimicrobicus* SMC 277^T^ has the potential to produce novel metabolites.

### Antibacterial activity of strain SMC 277^T^

To further explain the biosynthetic potential of strain SMC 277^T^, antimicrobial activity was assayed against target microoganisms including ESKAPE bacterial pathogens associated with nosocomial infections. The antimicrobial activity profile of strain SMC 277^T^ is shown in Table 3. The results indicated that the extract from strain SMC 277^T^ was able to inhibit the growth of several drug-sensitive bacteria, i.e., *S*. *aureus* DMST 8840, *K*. *pneumoniae* DMST 7592, *A*. *baumannii* DMST 10437 and *E*. *aerogenes* DMST 8841, and drug-resistant bacteria, i.e., MRSA strain AMH 10, ESBL producing *K*. *pneumoniae* AMH 20 and MDR *A*. *baumannii* AMH 30, when cultured for 7, 14 and 21 days. The inhibitory activity was not observed against *E*. *faecalis* DMST 2860 and *P*. *aeruginosa* DMST 4739. The largest zone of inhibition was observed against *A*. *baumannii* DMST 10437. These results indicated that strain SMC 277^T^ may prove a promising candidate to produce secondary metabolites with a wide capability of antimicrobial activities against bacterial pathogens.

**Table 3:** Antimicrobial activity of strain SMC 277^T^ measured by zone of inhibition against ESKAPE bacterial pathogens.

Bioassay microorganism	Diameter of inhibition zone (mm)		
	7 days	14 days	21 days
*Enterococcus faecalis* DMST 2860	-	-	-
*Staphylococcus aureus* DMST 8840	13.3 ± 0.6	12.3 ± 0.6	11.7 ± 0.6
*Klebsiella pneumoniae* DMST 7592	16.0 ± 1.0	13.3 ± 0.6	13.3 ± 0.6
*Acinetobacter baumannii* DMST 10437	19.0 ± 1.0	18.3 ± 0.6	17.7 ± 0.6
*Pseudomonas aeruginosa* DMST 4739	-	-	-
*Enterobacter aerogenes* DMST 8841	12.0 ± 1.0	10.7 ± 0.6	10.7 ± 0.6
Methicillin-resistant *Staphylococcus aureus* AMH 10	10.7 ± 0.6	9.7 ± 0.6	9.3 ± 0.6
Extended-spectrum β-lactamase producing *Klebsiella pneumoniae* AMH 20	13.3 ± 0.6	12.3 ± 0.6	12.3 ± 0.6
Multidrug-resistant *Acinetobacter baumannii* AMH 30	15.0 ± 1.0	12.3 ± 0.6	11.3 ± 0.6

Its extracts at various cultivation times (7, 14 and 21 days) were assayed for bioactivity against ESKAPE pathogenic bacteria. The diameter of zone of inhibition was expressed as mean ± standard deviation. -, no zone of inhibition.

## Conclusion

A novel *Streptomyces* strain with antibacterial activity, SMC 277^T^, was isolated from the clay soil in paddy field of Chonburi Province, Thailand. The 16S rRNA gene sequence similarity, mycelium and spore morphologies, and chemotaxonomic properties were comparable to those of validly published *Streptomyces* taxa. Differences in phenotypic characteristics (morphology on various cultivation media, and physiology) and low ORGI values (ANI values <95%, AAI values <95% and dDDH <70%) clearly distinguished strain SMC 277^T^ from its closest phylogenetic neighbor. Genome annotation and secondary metabolite gene cluster analysis revealed that strain SMC 277^T^ harbored NRPSs as the dominant classes of predicted BGCs and displayed 7 putative products as novel metabolites. This finding together with the observed antibacterial activity profile of the strain suggested that SMC 277^T^ is capable of producing bioactive metabolites against drug-resistant bacteria associated with nosocomial infections. Based on the analysis of polyphasic taxonomy in this study, they clearly distinguished strain SMC 277^T^ from the currently and validly published species of *Streptomyces*. Therefore, strain SMC 277^T^ should be recognized as representing a novel species of the genus *Streptomyces*, for which the name *Streptomyces antimicrobicus* sp. nov. is proposed. The species description of *S*. *antimicrobicus* sp. nov. is provided in Table 4.

**Table 4:** Description of *Streptomyces antimicrobicus* sp. nov.

Genus name	*Streptomyces*
Species name	*Streptomyces antimicrobicus*
Species epithet	*antimicrobicus*
Species status	sp. nov.
Species etymology	an.ti.mi.cro′bi.cus. Gr. Pref. anti-, against; N.L. neut. n. microbium, microbe; L. adj. suff. -icus -a -um, suffix used with various meanings; N.L. masc. adj. *antimicrobicus*, referring to antimicrobial
Description of the new taxon and diagnostic traits	Cells are aerobic, Gram-stain-positive actinomycete that form straight spore chains with a smooth surface on only aerial mycelium. Each spore size is 0.5–0.7 by 1.1–1.3 μm. Strain SMC 277^T^ grew well on ISP 2 and ISP 3, moderately on ISP 4, and poorly on ISP 5, ISP 6 and ISP 7. The color of substrate mycelium is pale yellow on ISP 2 and ISP 3, pale orange yellow on ISP 4, yellowish white on ISP 5 and ISP 7 and grayish greenish yellow on ISP 6. Aerial mycelium is yellowish white on ISP 2, pinkish gray on ISP 3 and ISP 4, grayish yellowish pink on ISP 5, greenish white on ISP 7 and none on ISP 6. No diffusible pigment was observed in cultures on all tested media. The growth temperature is between 20 and 40°C. The pH range for growth is 7–11. The maximum NaCl concentration for growth is 4% (w/v). Several substrates including D-arabinose, D-glucose, D-maltose, D-mannose, D-trehalose, glycerol, sucrose or salicin are used as sole carbon sources, but not D-fructose, D-galactose, D-lactose, D-mannitol, D-melibiose, D-raffinose, D-ribose, D-xylose, L-rhamnose, L-sorbose, arabitol, cellobiose, inositol, sorbitol or xylitol. Milk coagulation, and hydrolysis of starch, hypoxanthine and xanthine are positive, but gelatin liquefaction, milk peptonization and nitrate reduction are negative. According to the API-ZYM system, it displays activities of alkaline phosphatase, leucine arylamidase, valine arylamidase, trypsin, α-chymotrypsin, acid phosphatase, β-galactosidase, α-glucosidase, β- glucosidase, α-mannosidase, N-acetyl-β-glucosaminidase and naphthol-AS-BI-phosphohydrolase. Esterase (C4), esterase lipase (C8) and cystine arylamidase activities are weak. No activities are noted of lipase (C14), α-galactosidase, β- glucuronidase and α-fucosidase. The cell wall contains LL-diaminopimelic acid. The whole cell sugars are glucose, galactose, mannose and ribose. The predominant menaquinone is MK-9(H_8_) and MK-9(H_6_). Major cellular fatty acids (>10%) are iso-C_16:0_, anteiso-C_15:0_ and anteiso-C_17:0_. The profile of polar lipids contains phosphatidylglycerol, phosphatidylethanolamine, diphosphatidylglycerol, phosphatidylinositol, phosphatidylinositol mannoside and phospholipids.
Country of origin	Thailand
Region of origin	Chonburi Province
Source of isolation	Clay soil in paddy field
16S rRNA gene accession no.	OK380049
Genome accession no.	JAJAUY000000000
Genome status	Draft assembly
Genome size (bp)	6,545,928
DNA G+C content (mol%)	73.4
Strain collection numbers	= TBRC 15568^T^, = NBRC 115422^T^

## Supporting information

S1 Fig **a. Maximum parsimony tree based on 16S rRNA gene sequences showing the phylogenetic position of *Streptomyces antimicrobicus* SMC 277**^**T**^ **relative to the top 41 closely related species of the genus *Streptomyces*.** *Nocardioides albus* KCTC 9186^T^ was used as an outgroup. Numerals at nodes indicate bootstrap percentages derived from 1000 replications, and only values greater than 50% are indicated. **b. Maximum-likelihood tree based on 16S rRNA gene sequences showing the phylogenetic position of *Streptomyces antimicrobicus* SMC 277**^**T**^ **relative to the top 41 closely related species of the genus *Streptomyces*.** *Nocardioides albus* KCTC 9186^T^ was used as an outgroup. Numerals at nodes indicate bootstrap percentages derived from 1000 replications, and only values greater than 50% are indicated.
(PDF)

S2 Fig LL-diaminopimelic acid in the cell-wall peptidoglycan of *Streptomyces antimicrobicus* SMC 277^T^. (PDF)

S3 Fig The compositions of reducing sugar in whole-cell hydrolysates of *Streptomyces antimicrobicus* SMC 277^T^ analyzed using cellulose TLC. Its whole cell sugars consisted of glucose, galactose, mannose and ribose.
(PDF)

S4 Fig Polar lipid profiles of *Streptomyces antimicrobicus* SMC 277^T^ separated by 2-dimensional thin layer chromatography, and stained with phosphomolybdic acid (for detection of total lipids), Dittmer & Lester reagent (phospholipids), ninhydrin (amines), and anisaldehyde (sugars). Abbreviations: DPG, diphosphatidylglycerol; PG, phosphatidylglycerol; PE, phosphatidylethanolamine; PI, phosphatidylinositol; PIM, phosphatidylinositol mannoside; PL, phospholipid.
(PDF)

S1 Table The pairwise 16S rRNA gene sequence and overall genomic relatedness indices (ANIb, ANIm, AAI and dDDH values) of *Streptomyces antimicrobicus* SMC 277^T^ and the closest relative, *Streptomyces bambusae* NBRC 110903^T^ as well as other closely related type strains. (PDF)

S2 Table Morphological and physiological characteristics of *Streptomyces antimicrobicus* SMC 277^T^ and the closest relative, *Streptomyces bambusae* NBRC 110903^T^, as well as other closely related type strains. Strains: 1, SMC 277^T^; 2, *S*. *bambusae* NBRC 110903^T^; 3, *S*. *griseocarneus* DSM 40004^T^ (data from Reimer et al. [1], but some from Wen et al. [2] and Benedict et al. [3] as indicated by ^a^ and ^b^, respectively); 4, *S*. *coerulescens* NBRC 12758^T^ (data from Reimer et al. [1]); 5, *S*. *abikoensis* NBRC 13860^T^ (data from Reimer et al. [1], but some from Sujarit et al. [4] and Mingma et al. [5] as indicated by ^c^ and ^d^, respectively; 6, *S*. *yangpuensis* DSM 100336^T^ (data from Tang et al. [6]); 7, *S*. *virginiae* NBRC 12827^T^ (data from Reimer et al. [1], but some from Komaki et al. [7] as indicated by ^e^; 8, *S*. *amritsarensis* MTCC 11845^T^ (data from Sharma et al. [8], but some from Tang et al. [6] as indicated by ^f^; 9, *S*. *toxytricini* NBRC 12823^T^ (data from Reimer et al. [1], but some from Tamura et al. [9] as indicated by ^g^; 10, *S*. *cirratus* NBRC 13398^T^ (data from Reimer et al. [1]), but some from Koshiyama et al. [10] as indicated by ^h^. All data were generated in the present study unless indicated otherwise. +, positive; -, negative; w, weakly positive; N, No; Y, Yes; ND, not determined.
(PDF)

S3 Table Functional classification of protein-coding genes presented in genomes of *Streptomyces antimicrobicus* SMC 277^T^ and closely related type strains by the abundance of clusters of orthologous groups (COGs). Strains: 1, SMC 277^T^; 2, *S*. *bambusae* NBRC 110903^T^; 3, *S*. *toxytricini* NBRC 12823^T^; 4, *S*. *cirratus* NBRC 13398^T^; 5, *S*. *vinaceus* ATCC 27476^T^; 6, *S*. *nojiriensis* JCM 3382^T^; 7, *S*. *yangpuensis* DSM 100336^T^; 8, *S*. *virginiae* NBRC 12827^T^; 9, *S*. *amritsarensis* MTCC 11845^T^.
(PDF)

S4 Table Number and type of putative secondary metabolite biosynthesis gene clusters presented in various genomes of *Streptomyces antimicrobicus* SMC 277^T^ and closely related type strains. A hybrid cluster contains more than one type of secondary metabolite biosynthesis genes in the clusters. Strains: 1, SMC 277^T^; 2, *S*. *bambusae* NBRC 110903^T^; 3, *S*. *toxytricini* NBRC 12823^T^; 4, *S*. *cirratus* NBRC 13398^T^; 5, *S*. *vinaceus* ATCC 27476^T^; 6, *S*. *nojiriensis* JCM 3382^T^; 7, *S*. *yangpuensis* DSM 100336^T^; 8, *S*. *virginiae* NBRC 12827^T^; 9, *S*. *amritsarensis* MTCC 11845^T^.
(PDF)
