# Supplementary material for: Streptomyces antimicrobicus sp. nov., a novel clay soil-derived actinobacterium producing antimicrobials against drug-resistant bacteria
Source: PLoS One. 2023 May 31;18(5):e0286365. doi: 10.1371/journal.pone.0286365 (PMC10231761; doi:10.1371/journal.pone.0286365)

S1a Fig. Maximum parsimony tree based on 16S rRNA gene sequences showing the phylogenetic position of *Streptomyces antimicrobicus* SMC 277^{T} relative to the top 41 closely related species of the genus *Streptomyces*. *Nocardioides albus* KCTC 9186^{T} was used as an outgroup. Numerals at nodes indicate bootstrap percentages derived from 1000 replications, and only values greater than 50% are indicated.

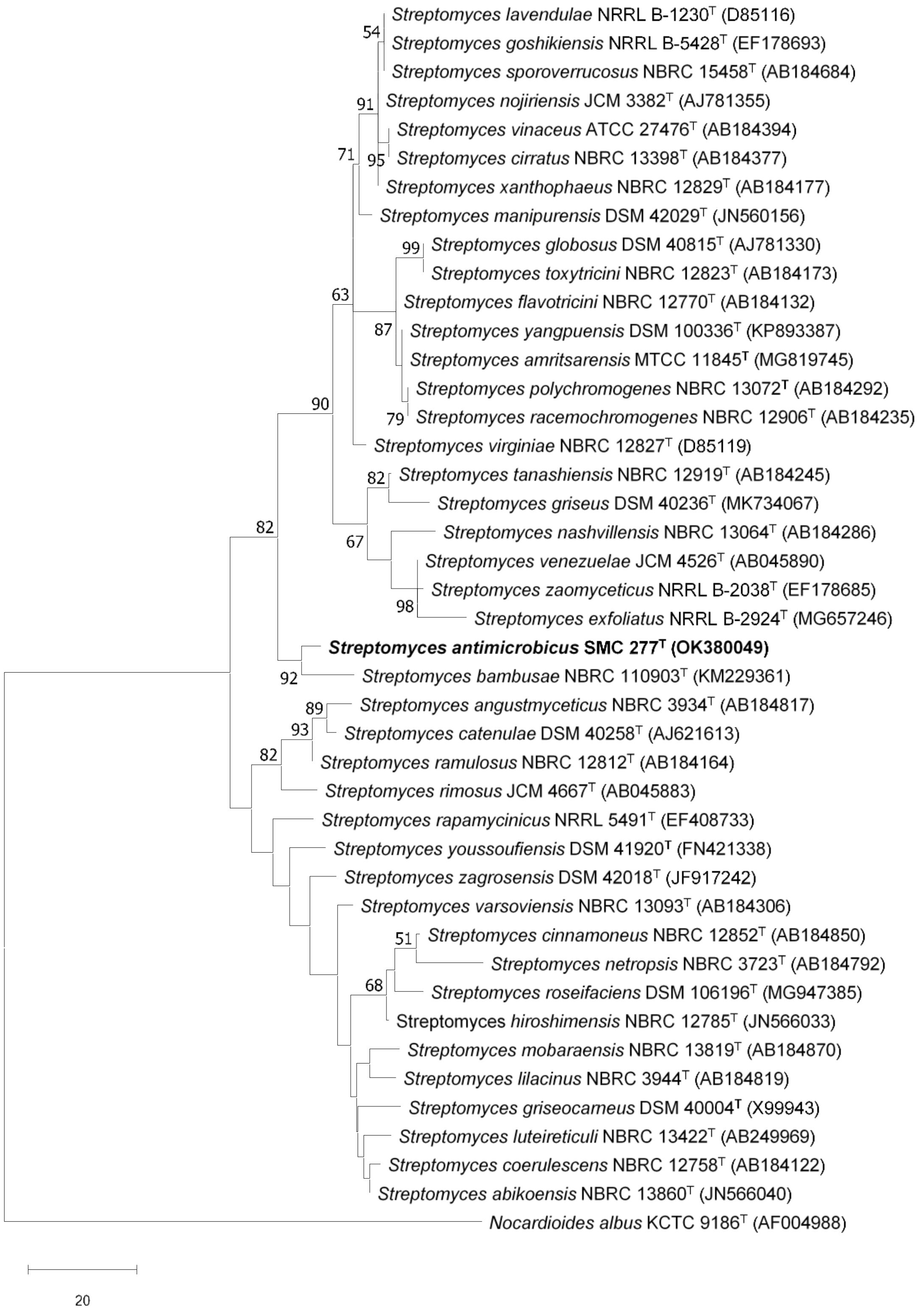

S1b Fig. Maximum-likelihood tree based on 16S rRNA gene sequences showing the phylogenetic position of *Streptomyces antimicrobicus* SMC 277^{T} relative to the top 41 closely related species of the genus *Streptomyces*. *Nocardioides albus* KCTC 9186^{T} was used as an outgroup. Numerals at nodes indicate bootstrap percentages derived from 1000 replications, and only values greater than 50% are indicated.

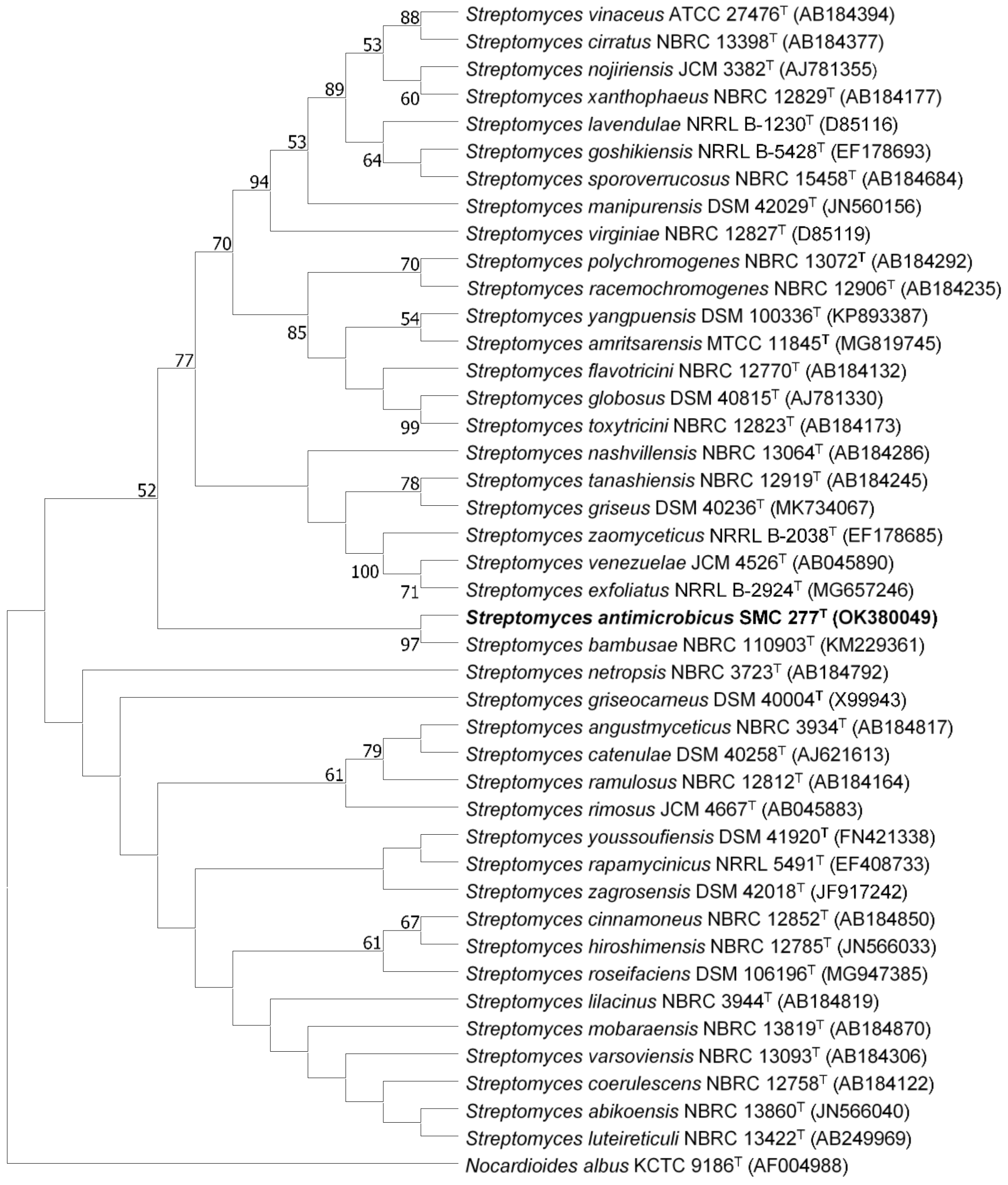

Supplement: S1 Fig — a. Maximum parsimony tree based on 16S rRNA gene sequences showing the phylogenetic position of Streptomyces antimicrobicus SMC 277T relative to the top 41 closely related species of the genus Streptomyces. Nocardioides albus KCTC 9186T was used as an outgroup. Numerals at nodes indicate bootstrap percentages derived from 1000 replications, and only values greater than 50% are indicated. b. Maximum-likelihood tree based on 16S rRNA gene sequences showing the phylogenetic position of Streptomyces antimicrobicus SMC 277T relative to the top 41 closely related species of the genus Streptomyces. Nocardioides albus KCTC 9186T was used as an outgroup. Numerals at nodes indicate bootstrap percentages derived from 1000 replications, and only values greater than 50% are indicated. (PDF) [file pone.0286365.s001.pdf]
