# Supplementary material for: Streptomyces antimicrobicus sp. nov., a novel clay soil-derived actinobacterium producing antimicrobials against drug-resistant bacteria
Source: PLoS One. 2023 May 31;18(5):e0286365. doi: 10.1371/journal.pone.0286365 (PMC10231761; doi:10.1371/journal.pone.0286365)

S2 Fig. LL-diaminopimelic acid in the cell-wall peptidoglycan of *Streptomyces antimicrobicus* SMC 277ᵀ.

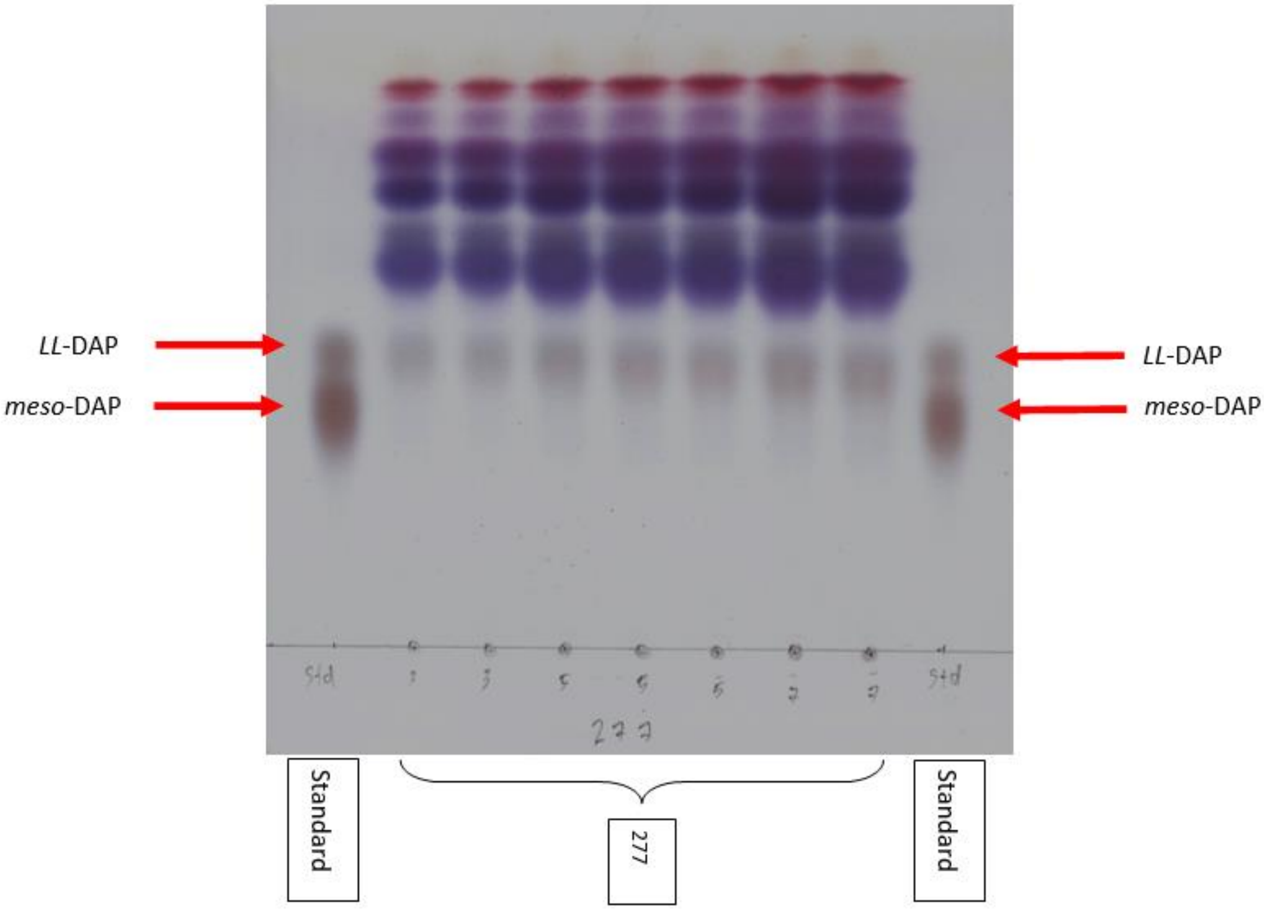

Supplement: S2 Fig — (PDF) [file pone.0286365.s002.pdf]
