# Supplementary material for: Streptomyces antimicrobicus sp. nov., a novel clay soil-derived actinobacterium producing antimicrobials against drug-resistant bacteria
Source: PLoS One. 2023 May 31;18(5):e0286365. doi: 10.1371/journal.pone.0286365 (PMC10231761; doi:10.1371/journal.pone.0286365)

S3 Fig. The compositions of reducing sugar in whole-cell hydrolysates of *Streptomyces antimicrobicus* SMC 277^{T} analyzed using cellulose TLC. Its whole cell sugars consisted of glucose, galactose, mannose and ribose.

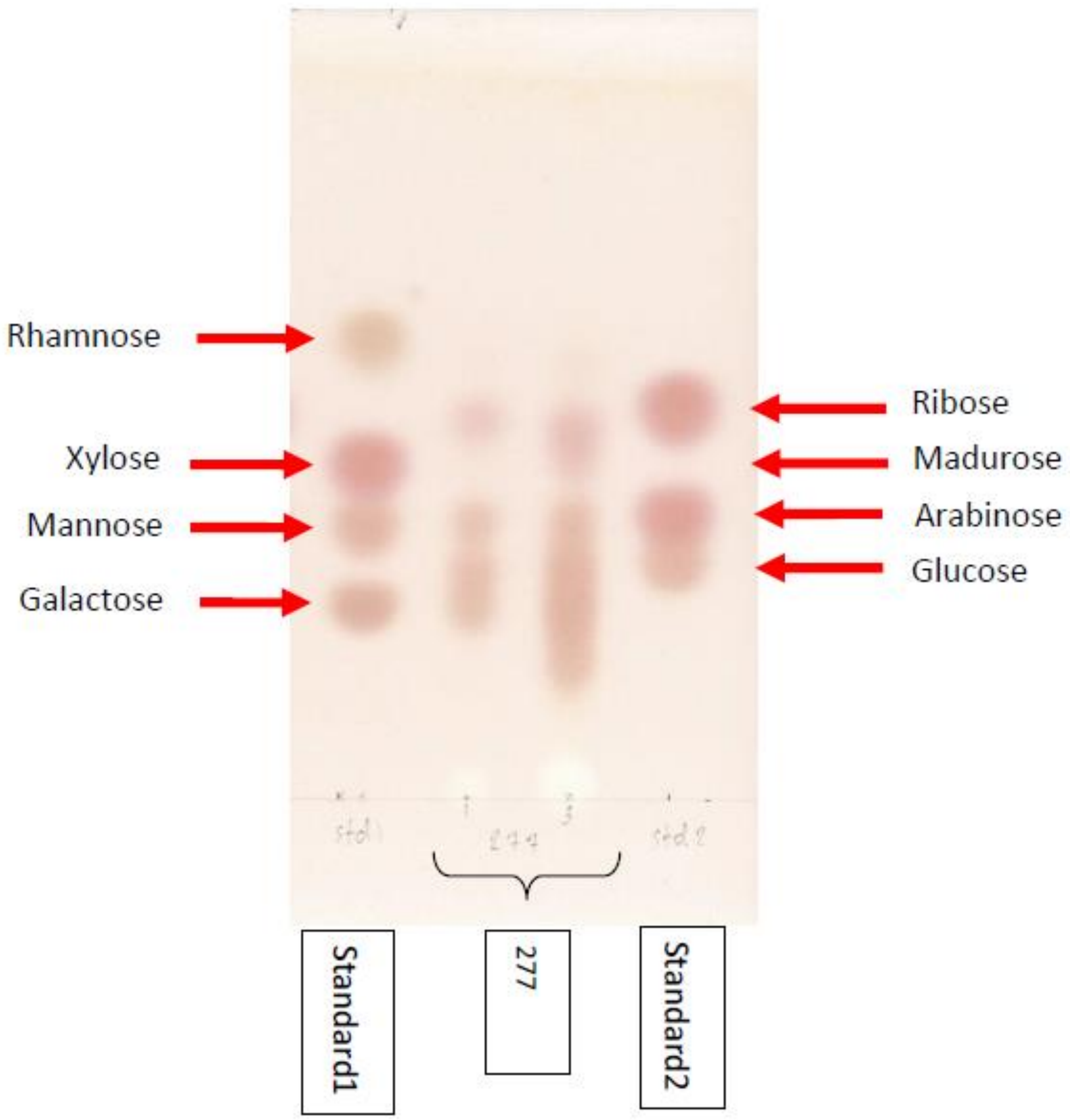

Supplement: S3 Fig — Its whole cell sugars consisted of glucose, galactose, mannose and ribose. (PDF) [file pone.0286365.s003.pdf]
