# Supplementary material for: Streptomyces antimicrobicus sp. nov., a novel clay soil-derived actinobacterium producing antimicrobials against drug-resistant bacteria
Source: PLoS One. 2023 May 31;18(5):e0286365. doi: 10.1371/journal.pone.0286365 (PMC10231761; doi:10.1371/journal.pone.0286365)
Supplement: S4 Fig — Abbreviations: DPG, diphosphatidylglycerol; PG, phosphatidylglycerol; PE, phosphatidylethanolamine; PI, phosphatidylinositol; PIM, phosphatidylinositol mannoside; PL, phospholipid. (PDF) [file pone.0286365.s004.pdf]

S4 Fig. Polar lipid profiles of *Streptomyces antimicrobicus* SMC 277^{T} separated by 2-dimensional thin layer chromatography, and stained with phosphomolybdic acid (for detection of total lipids), Dittmer & Lester reagent (phospholipids), ninhydrin (amines), and anisaldehyde (sugars). Abbreviations: DPG, diphosphatidylglycerol; PG, phosphatidylglycerol; PE, phosphatidylethanolamine; PI, phosphatidylinositol; PIM, phosphatidylinositol mannoside; PL, phospholipid.

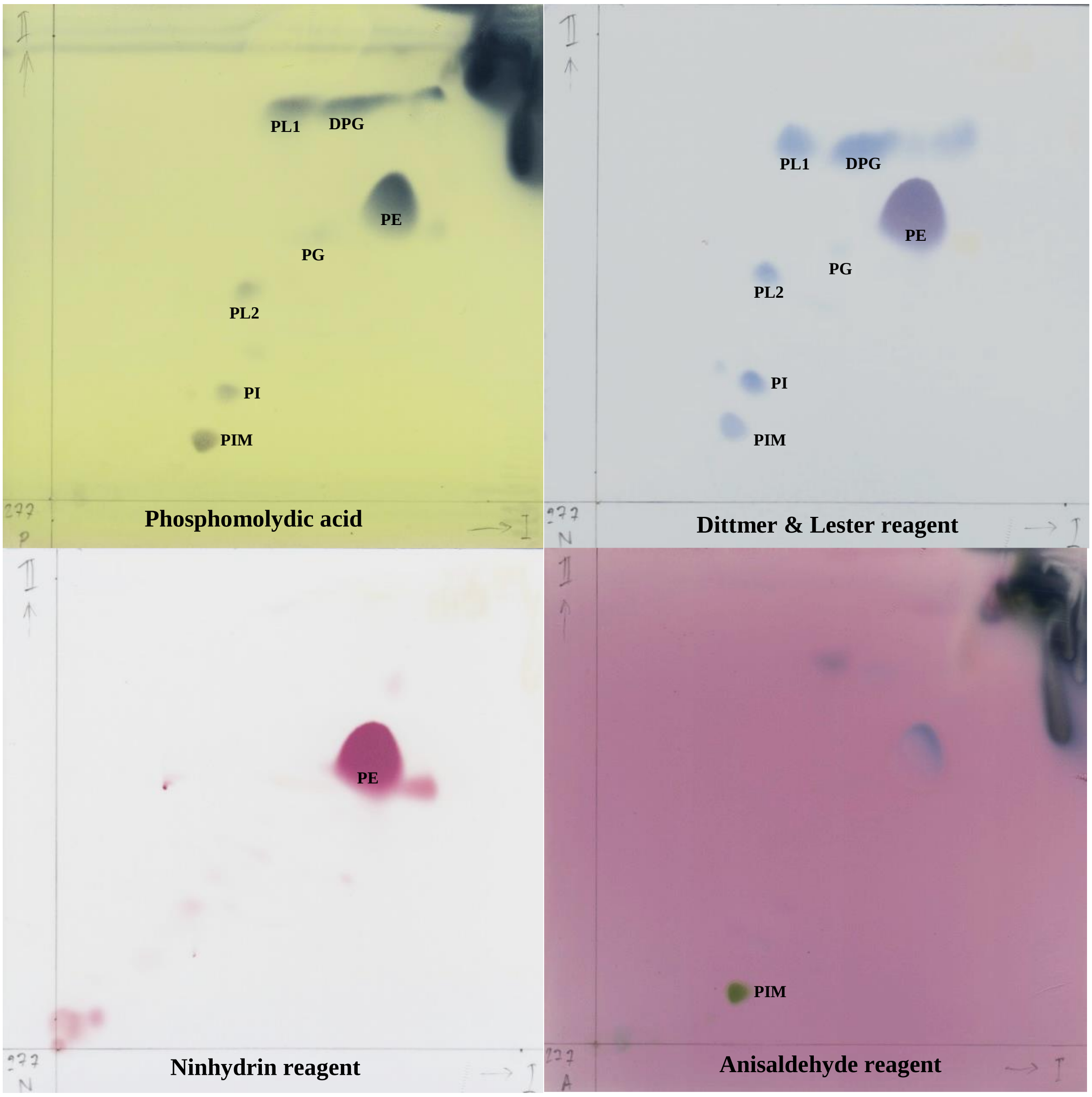

1st dimension →
2nd dimension ↑
