# Supplementary material for: Streptomyces antimicrobicus sp. nov., a novel clay soil-derived actinobacterium producing antimicrobials against drug-resistant bacteria
Source: PLoS One. 2023 May 31;18(5):e0286365. doi: 10.1371/journal.pone.0286365 (PMC10231761; doi:10.1371/journal.pone.0286365)
Supplement: S1 Table — (PDF) [file pone.0286365.s005.pdf]

S1 Table. The pairwise 16S rRNA gene sequence and overall genomic relatedness indices (ANIb, ANIm, AAI and dDDH values) of *Streptomyces antimicrobicus* SMC 277^{T} and the closest relative, *Streptomyces bambusae* NBRC 110903^{T} as well as other closely related type strains.

Strain	16S rRNA gene (%)	ANIb (%)	ANIm (%)	AAI (%)	dDDH (%) with C.I. model (%)
S. bambusae NBRC 110903^{T}	98.8	81.84	86.77	76.91	26.1 [23.7-28.6]
S. griseocarneus DSM 40004^{T}	98.4	77.77	85.72	70.20	23.1 [20.8-25.5]
S. abikoensis NBRC 13860^{T}	98.2	77.67	85.70	70.40	22.9 [20.6-25.4]
S. yangpuensis DSM 100336^{T}	98.2	82.86	86.70	80.20	26.9 [24.6-29.4]
S. roseifaciens DSM 106196^{T}	98.2	77.88	85.65	69.86	23.2 [20.9-25.7]
S. amritsarensis MTCC 11845^{T}	98.2	82.92	86.72	80.34	26.9 [24.5-29.4]
S. mobaraensis NBRC 13819^{T}	98.1	77.12	85.67	68.96	23.3 [21.0-25.7]
S. virginiae NBRC 12827^{T}	98.1	82.95	86.73	80.27	26.7 [24.4-29.2]
S. *angustmyceticus* NBRC 3934^{T}	98.0	77.82	85.58	69.96	23.0 [20.7-25.5]
S. hiroshimensis NBRC 12785^{T}	98.0	77.67	85.56	70.25	23.0 [20.7-25.5]
S. rimosus JCM 4667^{T}	97.9	77.57	85.45	69.43	23.0 [20.7-25.5]
S. catenulae DSM 40258^{T}	97.8	77.29	85.38	69.82	22.8 [20.5-25.3]
S. vinaceus ATCC 27476^{T}	97.8	82.91	86.75	80.36	26.8 [24.5-29.3]
S. lavendulae NRRL B-1230^{T}	97.8	82.87	86.69	79.93	26.7 [24.3-29.2]
S. nojiriensis JCM 3382^{T}	97.8	82.95	86.69	79.91	27.0 [24.6-29.4]
S. cirratus NBRC 13398^{T}	97.8	82.47	86.53	80.12	22.3 [23.9-28.7]
S. netropsis NBRC 3723^{T}	97.8	77.71	85.49	70.63	23.0 [20.7-25.5]
S. tanashiensis NBRC 12919^{T}	97.8	79.13	85.86	73.07	23.7 [21.4-26.1]
S. goshikiensis NRRL B-5428^{T}	97.8	82.91	86.68	79.81	26.7 [24.4-29.2]
S. zagrosensis DSM 42018^{T}	97.7	76.04	84.68	67.71	22.4 [20.2-24.9]
S. xanthophaeus NBRC 12829^{T}	97.7	82.83	86.60	80.08	26.7 [24.3-29.2]
S. cinnamoneus NBRC 12852^{T}	97.7	77.72	85.69	70.91	23.0 [20.7-25.4]
S. griseus DSM 40236^{T}	97.7	78.53	85.69	71.89	23.3 [21.0-25.8]
S. rapamycinicus NRRL 5491^{T}	97.6	77.02	85.15	68.65	22.5 [20.2-25.0]
S. varsoviensis NBRC 13093^{T}	97.6	77.36	85.29	69.68	23.0 [20.7-25.5]
S. venezuelae JCM 4526^{T}	97.6	79.01	85.91	72.94	23.6 [21.3-26.1]
S. zaomyceticus NRRL B-2038^{T}	97.6	78.83	85.84	73.06	23.4 [21.1-25.9]
S. exfoliatus NRRL B-2924^{T}	97.6	78.89	85.85	73.10	23.4 [21.1-25.8]
S. toxytricini NBRC 12823^{T}	97.6	82.31	86.71	79.61	26.3 [23.9-28.8]
S. nashvillensis NBRC 13064^{T}	97.6	79.08	85.88	73.09	23.6 [21.3-26.0]
