# Supplementary material for: Streptomyces antimicrobicus sp. nov., a novel clay soil-derived actinobacterium producing antimicrobials against drug-resistant bacteria
Source: PLoS One. 2023 May 31;18(5):e0286365. doi: 10.1371/journal.pone.0286365 (PMC10231761; doi:10.1371/journal.pone.0286365)
Supplement: S2 Table — Strains: 1, SMC 277T; 2, S. bambusae NBRC 110903T; 3, S. griseocarneus DSM 40004T (data from Reimer et al. [1], but some from Wen et al. [2] and Benedict et al. [3] as indicated by a and b, respectively); 4, S. coerulescens NBRC 12758T (data from Reimer et al. [1]); 5, S. abikoensis NBRC 13860T (data from Reimer et al. [1], but some from Sujarit et al. [4] and Mingma et al. [5] as indicated by c and d, respectively; 6, S. yangpuensis DSM 100336T (data from Tang et al. [6]); 7, S. virginiae NBRC 12827T (data from Reimer et al. [1], but some from Komaki et al. [7] as indicated by e; 8, S. amritsarensis MTCC 11845T (data from Sharma et al. [8], but some from Tang et al. [6] as indicated by f; 9, S. toxytricini NBRC 12823T (data from Reimer et al. [1], but some from Tamura et al. [9] as indicated by g; 10, S. cirratus NBRC 13398T (data from Reimer et al. [1]), but some from Koshiyama et al. [10] as indicated by h. All data were generated in the present study unless indicated otherwise. +, positive; -, negative; w, weakly positive; N, No; Y, Yes; ND, not determined. (PDF) [file pone.0286365.s006.pdf]

S2 Table. Morphological and physiological characteristics of *Streptomyces antimicrobicus* SMC 277^T and the closest relative, *Streptomyces bambusae* NBRC 110903^T, as well as other closely related type strains.

Strains: 1, SMC 277^T; 2, *S. bambusae* NBRC 110903^T; 3, *S. griseocarneus* DSM 40004^T (data from Reimer et al. [1], but some from Wen et al. [2] and Benedict et al. [3] as indicated by [a] and [b], respectively); 4, *S. coerulescens* NBRC 12758^T (data from Reimer et al. [1]); 5, *S. abikoensis* NBRC 13860^T (data from Reimer et al. [1], but some from Sujarit et al. [4] and Mingma et al. [5] as indicated by [c] and [d], respectively; 6, *S. yangpuensis* DSM 100336^T (data from Tang et al. [6]); 7, *S. virginiae* NBRC 12827^T (data from Reimer et al. [1], but some from Komaki et al. [7] as indicated by [e]; 8, *S. amritsarensis* MTCC 11845^T (data from Sharma et al. [8], but some from Tang et al. [6] as indicated by [f]; 9, *S. toxytricini* NBRC 12823^T (data from Reimer et al. [1], but some from Tamura et al. [9] as indicated by [g]; 10, *S. cirratus* NBRC 13398^T (data from Reimer et al. [1]), but some from Koshiyama et al. [10] as indicated by [h]. All data were generated in the present study unless indicated otherwise. +, positive; -, negative; w, weakly positive; N, No; Y, Yes; ND, not determined.

Characteristics	1	2	3	4	5	6	7	8	9	10
Morphology on culture medium:										
ISP 2										
Growth	Good	Good	Good	Good	Good	Good	Good	Good	Good	Good
Color of aerial mycelium	Yellowish white	Yellowish white	Grey	Pale green	Light grey	Sandy	White	Light grey	White	Cream
Color of substrate mycelium	Pale yellow	Grayish yellow	Brown	Brwon beige	Brown	Golden yellow	Beige	White	Light ivory	Brown beige
Diffusible pigment	N	N	N	N	Y	Y	N	Y	N	N
ISP 3										
Growth	Good	Good	Good	Good	Good	Good	Good	Good	Good	Good
Color of aerial mycelium	Pinkish grey	Greyish yellowish pink	White	Pale green	White	Sandy	White	Pinkish grey	Cream	Light grey
Color of substrate mycelium	Pale yellow	Greyish yellow	Brown	Brwon beige	Brown	Cream	Beige	White	Beige	Brown beige
Diffusible pigment	N	N	N	N	Y	N	N	N	N	N
ISP 4										
Growth	Moderate	Good	Good	Good	Good	Good	Good	Good	Good	Good
Color of aerial mycelium	Pinkish grey	Greyish yellowish pink	White	Pale green	White	Pale brown	Grey	Pinkish white	White	Light grey
Color of substrate mycelium	Pale orange yellow	Greyish yellow	Beige	Brwon beige	Brown	Beige	Beige	Pinkish white	Light ivory	Brown beige
Diffusible pigment	N	N	Y	N	Y	N	N	N	N	N
ISP 5										
Growth	Poor	Poor	Good	Good	Good	Poor	Good	Moderate	Good	Good

Color of aerial mycelium	Greyish yellowish pink	Greyish yellowish pink	Beige	None	Sparse	None	Grey	White	Cream	None
Color of substrate mycelium	Yellowish white	Greyish yellow	Brown	Beige	Brown	Pale yellow	Yellow	White	White	Beige
Diffusible pigment	N	N	Y	N	Y	N	N	N	N	N
ISP 6										
Growth	Poor	Poor	Good	Good	Good	Poor	Good	Good	Good	Good
Color of aerial mycelium	None	None	None	None	None	None	None	Brown	None	None
Color of substrate mycelium	Grayish greenish yellow	Dark greenish grey	Brown	Brown beige	Brown	Pale yellow	Colorless	White	Pale brown	Beige
Diffusible pigment	N	Y	Y	Y	Y	N	N	N	Y	N
ISP 7										
Growth	Poor	Moderate	Good	Good	Good	Moderate	Good	Good	Good	Good
Color of aerial mycelium	Greenish white	Greenish white	White	None	White	Light brown	Red	White	White	None
Color of substrate mycelium	Yellowish white	Light olive brown	Beige	Brown beige	Brown	Beige	Yellow	White	Light ivory	Brown beige
Diffusible pigment	N	N	N	N	Y	N	N	N	N	N
Growth at:										
Temperature (°C)	20-40	20-30	15-40[a]	ND	10-45[c]	10-40	15-37[e]	15-37[f]	15-37[g]	ND
pH	7-11	7-11	6-10[a]	ND	5-8[d]	5-10	6-9[e]	6-10[f]	6-10[g]	ND
Maximum tolerance to NaCl (%, w/v)	4	4	2	ND	5[d]	4	2	2	2[g]	ND
Hydrolysis of xanthine	+	-	ND	ND	ND	ND	ND	ND	ND	ND
Gelatin liquefaction	-	+	+	+	+	ND	-	-	w[g]	+
Milk coagulation	+	-	ND	ND	ND	ND	ND	ND	ND	ND
Milk peptonization	-	+	ND	ND	ND	ND	ND	ND	ND	ND
Nitrate reduction	-	+	ND	ND	ND	ND	+[e]	-	w[g]	-[h]
Carbon source utilization:										
D-Arabinose	+	w	-	+	-[d]	-	-[e]	-	w[g]	+
Arabitol	-	-	ND	ND	ND	ND	ND	ND	ND	ND
Cellobiose	-	+	-[a]	ND	-[d]	+	ND	+	ND	+[h]
D-Fructose	-	-	-	+	+	-	+	+	-[g]	+
D-Galactose	-	w	+[a]	ND	-	-	ND	-[f]	ND	+[h]
D-Glucose	+	+	+	+	+	+	+	+	+	+
Glycerol	+	+	ND	ND	ND		ND	ND	ND	ND
Inositol	-	-	+	+	-	-	-	-[f]	+[g]	-
D-Lactose	-	-	-[b]	ND	-[d]	-	-[e]	-[f]	-	-[h]

D-Maltose	+	+	+[b]	ND	ND	+	+[e]	ND	-	+[h]
D-Mannitol	-	w	-	+	-[d]	-	-	-[f]	-[g]	-
D-Mannose	+	+	ND	ND	ND	ND	ND	ND	ND	ND
D-Melibiose	-	-	ND	ND	ND	ND	ND	ND	ND	ND
D-Raffinose	-	-	-	+	-[d]	-	-[e]	-	-[g]	-
L-Rhamnose	-	-	-	+	-[d]	+	-	-	-[g]	-
D-Ribose	-	-	ND	ND	ND	-	ND	-[f]	-	ND
Salicin	w	w	+[b]	ND	ND	ND	+[e]	ND	ND	+[h]
Sorbitol	-	-	-[b]	ND	-	-	ND	-[f]	ND	-[h]
L-Sorbose	-	-	ND	ND	-	ND	-[e]	ND	ND	w[h]
Sucrose	+	+	-	+	-	-	-	w	-[g]	w[h]
Xylitol	-	-	-	ND	-[d]	ND	-	ND	ND	ND
D-Xylose	-	+	-	-	-	-	-	-	+[g]	-
D-Trehalose	w	-	ND	ND	ND	ND	ND	ND	ND	ND
Enzyme activity:										
Alkaline phosphatase	+	w	+	+	ND	ND	ND	ND	+	+
Esterase (C4)	w	w	+	+	ND	ND	ND	ND	+	+
Esterase lipase (C8)	w	w	+	+	ND	ND	ND	ND	+	+
Lipase (C14)	-	-	+	-	ND	ND	ND	ND	w	+
Leucine arylamidase	+	+	+	+	ND	ND	ND	ND	+	+
Valine arylamidase	+	+	+	+	ND	ND	ND	ND	+	+
Cystine arylamidase	w	-	+	-	ND	ND	ND	ND	w	+
Trypsin	+	+	+	+	ND	ND	ND	ND	+	+
α-Chymotrypsin	+	+	+	+	ND	ND	ND	ND	+	+
Acid phosphatase	+	+	+	+	ND	ND	ND	ND	+	+
Naphthol-AS-BI-phosphohydrolase	+	+	+	+	ND	ND	ND	ND	+	+
α-Galactosidase	-	-	-	+	ND	ND	ND	ND	-	-
β-Galactosidase	+	+	-	+	ND	ND	ND	ND	-	+
B-Glucuronidase	-	-	-	-	ND	ND	ND	ND	-	-
α-Glucosidase	+	-	+	+	ND	ND	ND	ND	-	+
β-Glucosidase	+	+	-	+	ND	ND	ND	ND	-	+
N-Acetyl- β-glucosaminidase	+	+	+	+	ND	ND	ND	ND	-	+
α-Mannosidase	+	-	+	+	ND	ND	ND	ND	+	+
α-Fucosidase	-	-	-	-	ND	ND	ND	ND	-	-

References

1. Reimer LC, Carbasse JS, Koblitz J, Ebeling C, Podstawka A, Overmann J. Bac*Dive* in 2022: the knowledge base for standardized bacterial and archaeal data**.** Nucleic Acids Res. 2022;50:D741-D746.
2. Wen Y, Zhang G, Bahadur A, Liu Y, Zhang Z, Chen T, et al. *Streptomyces gobiensis* sp. nov., an antimicrobial producing actinobacterium isolated from soil under black Gobi rocks. Int J Syst Evol Microbiol. 2022;72:005318.
3. Benedict RG, Lindenfelser A, Stodola FH, Traufler DH. Studies on *Streptomyces griseocarneus* and the production of hydroxystreptomycin. J Bacteriol. 1951;62:487-497.

4. Sujarit K, Kudo T, Ohkuma M, Pathom-Aree W, Lumyong S. *Streptomyces palmae* sp. nov., isolated from oil palm (*Elaeis guineensis*) rhizosphere soil. Int J Syst Evol Microbiol. 2016;66:3983-3988.
5. Mingma R, Duangmal K, Thamchaipenet A, Trakulnaleamsai S, Matsumoto A, takahashi Y. *Streptomyces oryzae* sp. nov., an endophytic actinomycete isolated from stems of rice plant. J Antibiot. 2015;68:368-372.
6. Tang B, Yu Y, Zhi X, Yang L, Cen X, Zhao G, et al. *Streptomyces yangpuensis* sp. nov., isolated from soil. Int J Syst Evol Microbiol. 2016;66:1224-1229.
7. Komoki H, Tamura T. Reclassification of *Streptomyces cinnamonensis* as a later heterotypic synonym of *Streptomyces virginiae*. Int J Syst Evol Microbiol. 2021;71:004813.
8. Sharma D, Mayilraj S, Manhas RK. *Streptomyces amritsarensis* sp. nov., exhibiting broad-spectrum antimicrobial activity. Antonie van Leeuwenhoek. 2014;105:943-949.
9. Tamura S, Nobutaka T, Miyamoto S, Mori R, Suzuki S, Nagatsu J. Isolation and physiological activities of piericidin A, a natural insecticide produced by *Streptomyces*. Agr Biol Chem. 1963;27:576-582.
10. Koshiyama H, Okanishi M, Ohmori T, Miyaki T, Tsukiura H, Matsuzaki M, et al. Cirramycin, a new antibiotic. J Antibiotics, Ser. A. 1963;16:59-66.
