# Supplementary material for: Streptomyces antimicrobicus sp. nov., a novel clay soil-derived actinobacterium producing antimicrobials against drug-resistant bacteria
Source: PLoS One. 2023 May 31;18(5):e0286365. doi: 10.1371/journal.pone.0286365 (PMC10231761; doi:10.1371/journal.pone.0286365)
Supplement: S3 Table — Strains: 1, SMC 277T; 2, S. bambusae NBRC 110903T; 3, S. toxytricini NBRC 12823T; 4, S. cirratus NBRC 13398T; 5, S. vinaceus ATCC 27476T; 6, S. nojiriensis JCM 3382T; 7, S. yangpuensis DSM 100336T; 8, S. virginiae NBRC 12827T; 9, S. amritsarensis MTCC 11845T. (PDF) [file pone.0286365.s007.pdf]

S3 Table. Functional classification of protein-coding genes presented in genomes of *Streptomyces antimicrobicus* SMC 277^T and closely related type strains by the abundance of clusters of orthologous groups (COGs).

Strains: 1, SMC 277^T; 2, *S. bambusae* NBRC 110903^T; 3, *S. toxytricini* NBRC 12823^T; 4, *S. cirratus* NBRC 13398^T; 5, *S. vinaceus* ATCC 27476^T; 6, *S. nojiriensis* JCM 3382^T; 7, *S. yangpuensis* DSM 100336^T; 8, *S. virginiae* NBRC 12827^T; 9, *S. amritsarensis* MTCC 11845^T.

Functions	1	2	3	4	5	6	7	8	9
General function prediction only	0.160	0.164	0.160	0.163	0.164	0.173	0.162	0.156	0.166
Transcription	0.129	0.132	0.133	0.136	0.139	0.148	0.137	0.130	0.134
Amino acid transport and metabolism	0.107	0.107	0.098	0.102	0.103	0.091	0.093	0.105	0.096
Signal transduction mechanisms	0.080	0.085	0.084	0.083	0.086	0.087	0.090	0.078	0.088
Function unknown	0.078	0.074	0.080	0.077	0.077	0.081	0.082	0.078	0.080
Carbohydrate transport and metabolism	0.074	0.076	0.072	0.080	0.078	0.068	0.068	0.072	0.072
Energy production and conversion	0.071	0.068	0.070	0.070	0.073	0.070	0.068	0.071	0.068
Lipid transport and metabolism	0.067	0.064	0.062	0.069	0.060	0.061	0.059	0.067	0.060
Secondary metabolites biosynthesis, transport and catabolism	0.051	0.052	0.047	0.057	0.044	0.052	0.046	0.075	0.047
Coenzyme transport and metabolism	0.050	0.048	0.051	0.047	0.046	0.045	0.045	0.048	0.048
Translation, ribosomal structure and biogenesis	0.048	0.046	0.050	0.046	0.044	0.043	0.046	0.049	0.045
Inorganic ion transport and metabolism	0.046	0.049	0.044	0.041	0.047	0.044	0.047	0.047	0.047
Cell wall/membrane/ envelope biogenesis	0.044	0.047	0.046	0.042	0.046	0.046	0.049	0.045	0.049
Replication, recombination and repair	0.035	0.039	0.039	0.039	0.039	0.042	0.042	0.044	0.040
Posttranslational modification, protein turnover, chaperones	0.030	0.031	0.034	0.030	0.033	0.030	0.032	0.028	0.032
Nucleotide transport and metabolism	0.026	0.024	0.027	0.025	0.024	0.025	0.025	0.024	0.023
Defense mechanisms	0.021	0.023	0.024	0.022	0.021	0.027	0.025	0.022	0.025

Intracellular trafficking, secretion, and vesicular transport	0.008	0.007	0.008	0.007	0.007	0.008	0.007	0.008	0.007
Cell cycle control, cell division, chromosome partitioning	0.008	0.008	0.008	0.008	0.007	0.007	0.009	0.008	0.008
Cell motility	0.001	0.001	0.001	0.001	0.001	0.001	0.001	0.002	0.001
RNA processing and modification	0.000	0.000	0.000	0.000	0.000	0.000	0.000	0.000	0.000
Cytoskeleton	0.000	0.001	0.001	0.001	0.001	0.001	0.001	0.000	0.000
Chromatin structure and dynamics	0.000	0.000	0.000	0.000	0.000	0.000	0.000	0.000	0.000
