# Supplementary material for: Streptomyces antimicrobicus sp. nov., a novel clay soil-derived actinobacterium producing antimicrobials against drug-resistant bacteria
Source: PLoS One. 2023 May 31;18(5):e0286365. doi: 10.1371/journal.pone.0286365 (PMC10231761; doi:10.1371/journal.pone.0286365)
Supplement: S4 Table — A hybrid cluster contains more than one type of secondary metabolite biosynthesis genes in the clusters. Strains: 1, SMC 277T; 2, S. bambusae NBRC 110903T; 3, S. toxytricini NBRC 12823T; 4, S. cirratus NBRC 13398T; 5, S. vinaceus ATCC 27476T; 6, S. nojiriensis JCM 3382T; 7, S. yangpuensis DSM 100336T; 8, S. virginiae NBRC 12827T; 9, S. amritsarensis MTCC 11845T. (PDF) [file pone.0286365.s008.pdf]

S4 Table. Number and type of putative secondary metabolite biosynthesis gene clusters presented in various genomes of *Streptomyces antimicrobicus* SMC 277^T and closely related type strains. A hybrid cluster contains more than one type of secondary metabolite biosynthesis genes in the clusters.

Strains: 1, SMC 277^T; 2, *S. bambusae* NBRC 110903^T; 3, *S. toxytricini* NBRC 12823^T; 4, *S. cirratus* NBRC 13398^T; 5, *S. vinaceus* ATCC 27476^T; 6, *S. nojiriensis* JCM 3382^T; 7, *S. yangpuensis* DSM 100336^T; 8, *S. virginiae* NBRC 12827^T; 9, *S. amritsarensis* MTCC 11845^T.

**BGC Types**	**1**	**2**	**3**	**4**	**5**	**6**	**7**	**8**	**9**
Hybrid clusters	10	5	12	9	8	9	6	8	7
betalactone	0	1	0	0	0	0	0	1	0
butyrolactone	1	1	0	0	0	1	2	1	1
CDPS	1	1	1	1	0	1	0	0	1
ectoine	0	0	0	1	0	0	0	0	0
indole	0	1	0	0	0	0	0	1	0
lanthipeptide class I	0	0	0	0	0	0	1	0	0
lanthipeptide class II	0	1	0	1	0	0	0	1	0
lanthipeptide class III	1	3	0	0	1	0	0	2	1
lanthipeptide class IV	0	0	1	0	1	2	1	0	0
LAP	0	1	0	0	0	0	0	0	0
lassopeptide	0	0	0	0	1	0	0	1	1
linaridin	0	0	1	0	1	0	0	1	0
melanin	2	3	0	0	1	1	1	0	1
NAPAA	0	0	0	2	1	0	0	0	1
NRPS	9	3	1	4	0	0	1	2	4
NRPS-like	2	1	0	0	0	1	2	1	2
nucleoside	0	1	0	0	0	0	0	0	1
PKS-like	0	0	0	1	0	0	0	0	0

RiPP-like	1	3	1	2	0	2	2	0	1
RRE-containing	0	0	0	0	0	0	0	1	0
siderophore	2	3	3	2	3	3	3	2	3
T1PKS	1	2	0	6	0	11	1	0	3
T2PKS	0	1	0	2	0	2	0	0	1
T3PKS	0	2	0	1	1	1	1	1	1
terpene	4	7	4	2	5	6	6	4	6
thiopeptide	1	0	0	0	0	0	0	0	0
**Total**	**35**	**40**	**24**	**34**	**23**	**40**	**27**	**27**	**35**

Abbreviation in S4 Table:

CDPS : tRNA-dependent cyclodipepetide synthases

LAP : Linear azo(in)e-containing peptides

NAPAA ; non-alpha poly-amino acids like e-Polylysin

NRPS : Non-ribosomal peptide synthetase cluster

NRPS-like : NRPS-like fragment

PKS-like : Other types of PKS cluster

RiPP-like : Other unspecified ribosomally synthesized and post-translationally modified peptide product (RiPP) cluster

RRE-containing : RRE-element containing cluster

T1PKS : Type I PKS (Polyketide synthase0

T2PKS : Type II PKS

T3PKS : Type III PKS
